# The RNA-Binding Domain NS1 of Influenza as an Antiviral Target: From Evolutionary Conservation Mapping to Experimental Validation

**DOI:** 10.3390/v18030279

**Published:** 2026-02-25

**Authors:** Luis André Santos, João Trigueiro-Louro, Helena Rebelo-de-Andrade

**Affiliations:** 1 Antiviral Resistance Lab, Research & Development Unit, Infectious Diseases Department, Instituto Nacional de Saúde Doutor Ricardo Jorge, IP, Av. Padre Cruz, 1649-016 Lisbon, Portugal; luis.santos@insa.min-saude.pt (L.A.S.); jmtlouro@gmail.com (J.T.-L.); 2 Host-Pathogen Interaction Unit, Research Institute for Medicines (iMed.ULisboa), Faculty of Pharmacy, Universidade de Lisboa, Av. Professor Gama Pinto, 1649-003 Lisbon, Portugal; 3 Hospital CUF Tejo Hospital, Av. 24 de Julho 171A, 1350-352 Lisbon, Portugal; 4 Serviço de Medicina, Hospital de São Francisco Xavier, Centro Hospitalar Lisboa Ocidental (CHLO), Estrada do Forte do Alto do Duque, 1449-005 Lisbon, Portugal

**Keywords:** influenza A virus, antiviral target, druggable pockets, mutagenesis, viral replication, viral fitness

## Abstract

Non-structural protein 1 (NS1) of influenza A virus is a multifunctional virulence factor and represents a promising anti-influenza target, considering its conserved and druggable structure. As antiviral target, NS1-RNA-binding domain (RBD) remains unexplored, despite its critical role in replication. In this study, we applied a “Map-and-Mutate” strategy to identify and functionally validate highly conserved and druggable regions within the NS1-RBD. Using large-scale sequence alignments and structural characterization, we integrated conservation and druggability analyses to predict conserved druggable pockets and top-ranked hot spots, mutate the five most promising residues (L15, W16, R19, R35, and L43) and study their impact on viral fitness. In vitro, the mutations W16 and R35 caused most significant reduction in viral fitness; however, L15 and R19 also impaired replication. Combined mutations involving W16 and either L15 or L43 exerted a cumulative effect, reducing viral replication, hemagglutination titers and neuraminidase activity. This study demonstrates that most residues identified and investigated using the “Map-and-Mutate” strategy negatively impact viral fitness, underscoring the approach’s value in pinpointing novel antiviral targets. Together with our prior research, this study reinforces the importance of NS1 as a promising antiviral target, providing a rationale for designing and developing therapies with a higher resilience to viral resistance.

## 1. Introduction

Influenza viruses continue to be a significant threat to global public health, causing seasonal epidemics of varying magnitude that affect millions of people worldwide. It is estimated that up to 20% of the population is infected with seasonal influenza each year [1,2]. Severe outcomes of influenza infection can result in hospitalization or death, especially in high-risk groups like the elderly, very young children, pregnant women, and individuals with chronic health conditions [3]. Influenza imposes a substantial economic burden, straining healthcare resources and leading to significant losses in global productivity [4].

Despite the availability of vaccines and classic antivirals, influenza’s evolution due to the rapid mutation rate challenges existing treatments and highlights the need for novel therapeutic approaches [5,6,7].

Current antiviral treatments for influenza, such as neuraminidase inhibitors and polymerase inhibitors, have shown efficacy in reducing the severity and duration of symptoms [8]. However, the emergence of antiviral resistance poses a major challenge, as resistant strains can compromise the effectiveness of current treatments [7,9,10]. Moreover, these drugs target viral structural components. Neuraminidase inhibitors target a mutable surface antigen, increasing the likelihood of resistance development. The PA subunit of polymerase, targeted by baloxavir, has also evidenced the emergence of resistance mutations following antiviral use, posing a significant challenge.

Therefore, there is a pressing need for antivirals with alternative mechanisms of action, especially those targeting more conserved proteins of the virus that are less likely to mutate over time.

In this regard, non-structural protein 1 (NS1) of the influenza virus presents a unique advantage. Unlike the surface glycoproteins hemagglutinin and neuraminidase (NA), which frequently mutate to evade host immune responses, the NS1 protein contains conserved regions that remain stable across diverse influenza strains [10,11,12].

Structurally, NS1 is divided into two main domains: the N-terminal RNA-binding domain (RBD), associated with structure dimerization and double-stranded RNA interaction, and the effector domain (ED), a multifunctional domain that interacts with multiple host factors to suppress innate/adaptative antiviral responses [13].

In this context, NS1 conserved sites are essential for its structure and functional roles in modulating the host immune response and promoting viral replication. Targeting these conserved sites with specific inhibitors could potentially disrupt NS1’s function, preventing the virus from evading immune host response and limiting viral replication [14,15]. Moreover, because these conserved regions are presumed to be critical for the virus’s replication and pathogenicity, they are less tolerant to mutations. This suggests that putative drugs targeting such sites could maintain efficacy longer than current antivirals aimed at structural proteins.

Our group previously conducted a comprehensive in silico analysis of the NS1 protein, focusing on the conservation and druggability of its ED, as well as the conservation of the RBD. This study was based on an extensive large-scale sequence analysis and structural characterization of NS1 across human-infecting influenza A subtypes spanning more than a century [16].

In addition, we identified three highly conserved and druggable pockets (Conserved Druggable Pockets, CDPs) within the NS1 effector domain (NS1-ED), which are amenable to pharmacological modulation. To validate our previous in silico findings, we experimentally assessed whether single amino acid (aa) mutations at selected hot spot residues within these CDPs would affect viral replication. Using influenza A(H1N1)pdm09 as a model, we found that mutations at positions 102, 121, 171, 175, and 184 significantly impaired viral replication in vitro. These residues therefore represent promising targets for antiviral intervention under the “Map-and-Mutate” strategy [15,16].

The aim of the present study is to apply the “Map-and-Mutate” strategy to the RNA-binding domain of the influenza virus NS1 protein (NS1-RBD) in order to identify novel targets for antiviral strategies. By integrating analyses of sequence conservation, structural features, and functional relevance, we seek to pinpoint the key regions that are critical for viral fitness. This approach may help to uncover antiviral targets that are not only effective, but also less prone to resistance due to the virus’s high mutation rate.

## 2. Materials and Methods

### 2.1. Dataset Construction and Conservation Analysis

The conservation data used in this study was previously performed by Trigueiro-Louro, 2019 [16]. Briefly, in this analysis, we included NS1 sequences of worldwide circulating viruses comprising 28,392 NS1 protein sequences from human-infecting influenza A viruses over more than 100 years (1918–2018), including strains originating from avian-to-human transmission events, for a total of 27,566 influenza A virus isolates previously established in the human population (H1N1, H1N2, H2N2 and H3N2) and 826 influenza A virus isolates of avian origin (H5N1, H5N6, H7N7, H7N9 and H9N2). Sequences were obtained from the NCBI Influenza Virus database at https://www.ncbi.nlm.nih.gov/genomes/FLU/Database/nph-select.cgi (accessed on 15 January 2026), GISAID’s EpiFlu™ database at https://www.gisaid.org (accessed on 15 January 2026) and Influenza Research Database, currently included in the Bacterial and Viral Bioinformatics Resource Center (BV-BRC—https://www.bv-brc.org (accessed on 15 January 2026) [17,18,19,20].

Amino acid conservation was evaluated using the Jalview AACons server and the Valdar scoring algorithm, following Trigueiro-Louro, 2019 [16]. Residues were scored from 0 (highly variable) to 11 (highly conserved), with scores ≤ 4 classified as variable.

### 2.2. Protein Structure

Three-dimensional structures of the influenza NS1-RBD protein were obtained from the RCSB Protein Data Bank (www.rcsb.org (accessed on 15 January 2026)) [21]. Structures of the influenza A/H1N1 NS1 protein were selected for analysis: the crystal structures with PDB entries 2ZKO [22] and 3M8A [23] and the Nuclear Magnetic Resonance (NMR) structures 1NS1 [24] and 2N74 [25]. The three-dimensional structures were accurately prepared (including removing ligands, protonation and performing energy minimization) using the Molecular Operating Environment (MOE) software (version 2024, Chemical Computing Group, Montreal, QC, Canada).

### 2.3. Druggability and Pocket Identification

The druggable sites in the RNA-binding domain of NS1 protein were predicted using the webservers DoGSiteScorer (DGSS, https://proteins.plus (accessed on 15 January 2026) and PockDrug-Server (PDS—http://pockdrug.rpbs.univ-paris-diderot.fr/ (accessed on 15 January 2026), as previously described in Trigueiro-Louro et al., 2019 [16].

The DGSS server employs a grid-based methodology integrated into a support vector machine (SVM) model. It evaluates potential binding pockets using a combination of geometric (e.g., size and shape) and physicochemical descriptors, assigning a druggability score ranging from 0 (undruggable) to 1 (highly druggable). In this study, pockets with scores equal to or above 0.4 were classified as druggable, following the criteria proposed by Volkamer et al., 2012 [26].

The PDS tool applies a geometry-driven strategy that integrates key structural features, including hydrophobicity and aromaticity, to estimate druggability. Pockets achieving a score ≥ 0.5 were considered druggable, in accordance with the methodology described by Hussein et al., 2015 [27].

A comprehensive integrative analysis was conducted to consolidate the druggability data obtained from both bioinformatic platforms for the NS1-RBD in the dimeric state (Supplemental Data Table S1). In the final stage of the study, a comparative assessment was carried out to identify the most consistently predicted druggable sites across the two tools. Druggability consensus for each group was cross-referenced with residue conservation data, enabling the identification of consensus druggable pockets (CDPs) and top-ranked hot spot (T-RHS) residues that spatially overlap with conserved regions. Notably, particular attention was given to residues corresponding to conserved sites in the NS1-RBD protein due to their functional relevance and potential cross-viral targeting.

### 2.4. Cells Lines

Madin Darby Canine Kidney cells with overexpression of the α-2,6-linked sialic acid receptor (MDCK-SIAT1, European Collection of Cell Cultures—ECACC, Salisbury, UK) support robust replication of human influenza A viruses and are widely employed for virus isolation and propagation, providing a consistent system for comparative fitness analyses [28,29]. These cells were maintained as previously described [30].

293T cells were maintained in growth medium (DMEM (Gibco, Paisley, UK) supplemented with 10% FBS (Gibco, Paisley, UK), 2 mM L-Glutamine (Gibco, Paisley, UK), 1 × NEAA (Gibco, Paisley, UK), 24 mM HEPES (Gibco, Paisley, UK), 2.5 μg/mL Fungizone (Gibco, Paisley, UK), and 1 × Penicillin-Streptomycin-Neomicin antibiotic mixture (PSN, Gibco, Paisley, UK)) and incubated at 37 °C in a humidified atmosphere of 5% (*v*/*v*) CO_2_.

### 2.5. Virus, Plasmids, and Mutagenesis

The complete gene library of A/Portugal/82/2009 (used as an A(H1N1)pdm09 representative strain), herein called PT82, was assembled in pCIPolISapIT, as previously described [31].

Residues identified as T-RHS—either overlapping with or located in close spatial proximity to conserved regions within the NS1-RBD, as determined by the in silico analysis—were selected for site-directed mutagenesis. Mutations in the NS1 plasmid sequence were introduced using the QuikChange II site-directed mutagenesis kit (Agilent, Cedar Creek, TX, USA) according to the manufacturer’s protocol. Nucleotide substitutions encoding for the NS1 single aa mutations L15A, W16A, R19A, R35A, L43A, L43R, the double aa mutations L15A + W16A, L15A + R35A, W16A + R35A, W16A + L43A, R35A + L43A, and the triple aa mutations L15A + W16A + R35A and W16A + R35A + L43A were introduced in the NS1 gene (rationale explained in Section 3.2). The correct sequence of each NS1 clone was confirmed by DNA sequencing using the CDC reference protocol recommended by the WHO [32,33].

### 2.6. Reverse Genetics

Wild-type PT82 (wt) and NS1 mutated viruses (L15A, W16A, R19A, R35A, L43A, L43R, L15A + W16A, L15A + R35A, W16A + R35A, W16A + L43A, R35A + L43A, L15A + W16A + R35A and W16A + R35A + L43A) were generated using the eight-plasmid-based reverse genetics system following an adaptation of the Hoffman method [34] previously described in Santos et al. [35]. The virus stocks were titrated by the plaque-forming units (PFU) titration assay (see Section 2.8) and stored at −80 °C.

### 2.7. In Vitro Infection

MDCK-SIAT1 cells were seeded at density of 2 × 10^4^ cells/cm^2^ in growth medium, in six-well plate, and incubated at 37 °C in a humidified atmosphere of 5% (*v*/*v*) CO_2_ for 48 h prior to infection. The wt and the NS1-mutated viruses were inoculated at a same multiplicity of infection (MOI) of 0.01 PFU/cell, in four independent assays. Supernatants of each experiment were collected at T12, T18, T24, T36 and T48 h post-infection (hpi), centrifuged at 400× *g* for 5 min, and stored at −80 °C [15,36].

### 2.8. Plaque-Forming Units Titration

The plaque-forming units titration assay was used, as previously described [15,37], to determine infectious viral titers of the recombinant virus’s stocks and of the collected time point supernatants. Virus titers were calculated as the number of plaque-forming units per millilitre.

### 2.9. MUNANA Neuraminidase Activity Assay

NA activity was determined for each time point and independent assay by an in-house fluorescence MUNANA (2′-(4-Methylumbelliferyl)-α-D-N-acetylneuraminic acid sodium salt hydrate)-based assay, adapted from the Public Health England (PHE, London, UK) protocol [38,39]. A cutoff of 12,500 relative fluorescence units (RFU) was established, and NA activity values were determined as the virus dilution at which this cutoff was reached.

### 2.10. Hemagglutination Assay

The hemagglutination (HA) titer was determined as previously described [35] using Guinea-Pig Red Blood Cells. The HA titer was defined as the reciprocal of the highest serum dilution that exhibited complete hemagglutination.

### 2.11. Statistical Analysis

For data and statistical analysis, titers less than 1 in HA and NA activity assays were defined as 1. Data was normalized with log_10_ (PFU titers) or log_2_ (HA and NA assays). The data was analyzed in R (Studio Team, 2019, RStudio: Integrated Development for R, Boston, MA, USA, http://www.rstudio.com/ (accessed on 15 January 2026)) using the appropriate packages. The normality and homogeneity of variances were evaluated by Levene’s and Shapiro–Wilk tests, respectively. Analysis of variance (ANOVA) was used to analyze the differences among group means in a sample, and Tukey’s range test was applied to perform single-step multiple comparisons between samples (wt and NS1-mutated viruses). Values are expressed as means plus or minus the standard error of the mean (Mean ± SEM). The threshold for statistical significance was established as *p* < 0.05, with a 95% confidence interval [15,36,37].

## 3. Results

### 3.1. The Influenza NS1-RBD Exhibits Conserved Druggable Pockets and Top-Ranked Hot Spots

A linear schematic representation of the whole NS1-RBD druggability was generated. This analysis was initially performed by merging the druggability information from the two bioinformatic tools (DGSS and PDS) for each NS1-RBD dimer structure. The potential druggable regions or sites are coloured in light blue and dark blue according to the druggability score scale (Supplementary Data Table S1).

**Figure 1:**
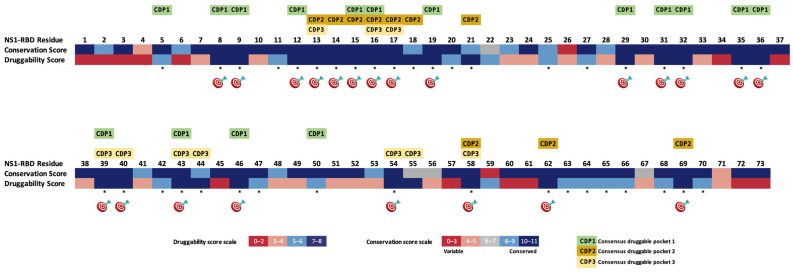
Overall alignment of NS1-RBD druggability and conservation scores for each residue position with the identification of a consensus druggable pocket. The druggability prediction was based on the descriptors algorithm of each pocket bioinformatics tool: DGSS and PDS. The potential conserved druggable regions/sites are marked with an asterisk and the top-ranked hot spots are marked with a target. The conserved druggable pockets (CDP1, CDP2 and CDP3) are also highlighted.

In a later phase of the study, a comprehensive comparison of NS1-RBD druggability, integrating the consensus of the druggable sites identified across all NS1-RBD models analyzed via the dual bioinformatic strategy (DGSS and PDS), along with corresponding residue conservation data, was conducted, as shown in Figure 1.

The conserved druggable regions/sites are marked with an asterisk and the T-RHS are marked with a target (Figure 1). The T-RHS for drug targeting identified in the global final analysis consisted of the following twenty-two residues: S8, F9, D12-H17, R19, D29, P31-F32, R35-L36, D39-Q40, L43, R46, I54, T58, K62, and L69.

Some of these regions are placed close together so that they can form larger pockets. In this regard, three main consensus pockets were described based on the comparative study of NS1-RBD druggability along with the degree of residue conservation (which were named as “consensus druggable pockets”—CDPs). Only pockets comprising more than 10 aa and with a high druggability score were considered.

The consensus druggable pocket 1 (CDP1) found among all NS1-RBD conformations comprises the following residues: 5, 8–9, 12, 15–16, 19, 29, 31–32, 35–36, 39, 43, 46, and 50. The consensus druggable pocket 2 (CDP2) comprises the following residues: 13–18, 21, 58, 62, and 69. The consensus druggable pocket 3 (CDP3) includes the residues 13, 16–17, 39–40, 43–44, 54–55, and 58.

**Figure 2:**
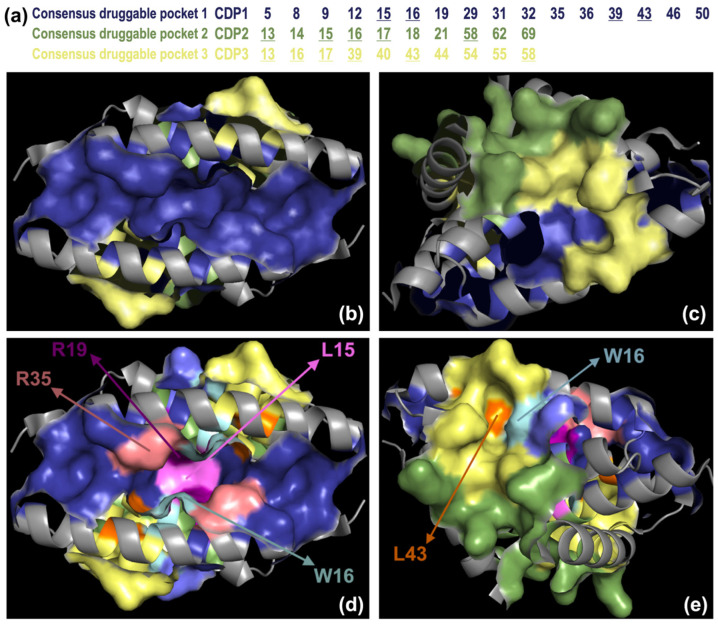
Mapping results of the predicted druggable pockets (**a**–**c**) and of key hot spots (**d**,**e**) onto the NS1-RBD crystallographic structure (PDB ID: 2ZKO). Residues belonging to each Consensus Druggable Pocket (CDP) are summarized in the table, residues present in more than one CDP are underlined. CDP1, CDP2 and CDP3 are coloured in blue, green and yellow, respectively (**a**–**e**). Top-ranked hot spot residues (L15, W16, R19, R35 and L43) are colour-coded for clarity: L15 (violet), W16 (cyan), R19 (purple), R35 (salmon) and L43 (orange). Molecular graphics were generated using the PyMOL Molecular Graphics System (Version 3.1.5.1, Schrödinger, LLC, New York, NY, USA).

Figure 2a–c displays the spatial arrangement of the CDPs along with the residues included in each pocket.

### 3.2. From in Silico to In Vitro

Following the completion of our in silico study, we deemed it essential to experimentally assess the impact of mutations at the prioritized sites on viral replication, thereby providing empirical validation of the computational predictions. Experimental data is essential to support the hypotheses generated using in silico approaches, which, while accelerating drug discovery and guiding research, benefit from intrinsically direct biological confirmation [40,41].

Residues identified as conserved and druggable T-RHS—namely L15 (CDP1 and CDP2), W16 (CDP1, CDP2, CDP3), R19 (CDP1), R35 (CDP1), and L43 (CDP1 and CDP3)—were selected for detailed in vitro investigation, given their potential to disrupt protein function through pocket(s) interactions. The spatial distribution and location of these residues within the CDPs and in the NS1-RBD structure are shown in Figure 2d,e. Our objective was to determine whether single aa mutations at these key positions within the NS1-RBD could impair viral replication, using the influenza A(H1N1)pdm09 strain as a model. This approach aimed to validate the potential of these sites as antiviral targets under the map-and-mutate strategy.

We evaluated viral fitness by measuring infectious viral titers (PFU), HA titer, and NA activity in NS1-mutant viruses relative to the wt control. Additionally, double and triple mutations were introduced at combinations of sites showing the most promising preliminary effects—specifically L15A + W16A, L15A + R35A, W16A + R35A, W16A + L43A, R35A + L43A, L15A + W16A + R35A, and W16A + R35A + L43A—to explore potential synergistic impacts on viral replication. The R19A mutation, which demonstrated less pronounced effects, was excluded from these multiple-mutant constructs (results are presented in Section 3.3, Section 3.4 and Section 3.5).

Given the unexpected and disappointing/unsatisfactory results observed with the L43A mutation (results are presented in Section 3.3, Section 3.4 and Section 3.5), and considering the critical functional role of residue L43, we performed an additional substitution to L43P. While the L43A mutation involves a conservative change, replacing leucine with a smaller, hydrophobic alanine that generally preserves physicochemical properties and causes minimal structural perturbation, the L43P mutation introduces a bulkier, positively charged arginine side-chain. This alteration is expected to induce more significant structural and functional changes, providing further insight into the importance of this residue.

### 3.3. Mutation of the Hot Spot Amino Acids Affects Viral Replication In Vitro

**Figure 3:**
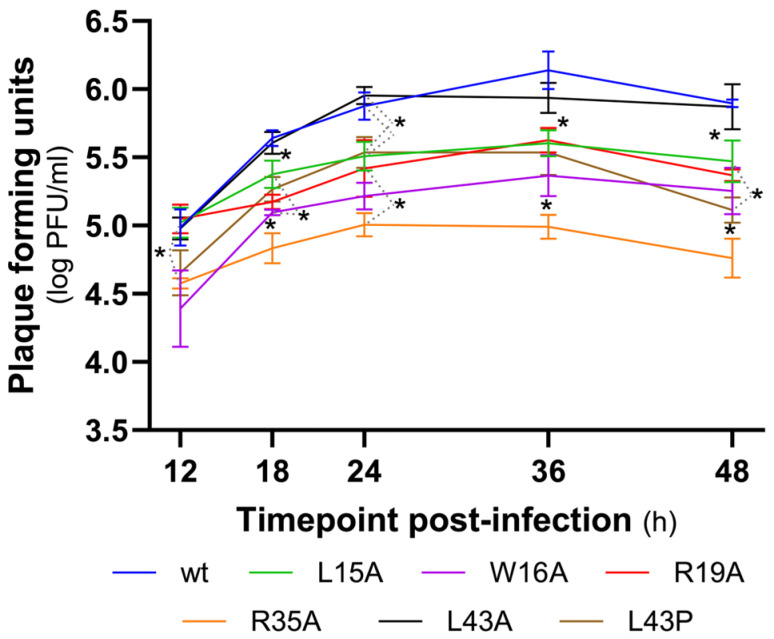
Kinetic replication curves of wt and single-mutated viruses. The infectious viral titer (PFU) was determined by avicel plaque assay at each time point post-infection (from T12 to T48) in four independent assays (*n* = 4). All values are expressed in log PFU/mL. Asterisks indicate statistically significant differences (*p* < 0.05 Tukey).

Plaque assays were performed to determine the infectious virus titer as plaque-forming units per millilitre (PFU/mL) at different time points (T12, T18, T24, T36 and T48) post-infection (hpi) in order to understand whether the NS1 single aa changes could affect viral replication (Figure 3 and Supplementary Data Table S2).

The studied viruses showed an increase in replication in the first 24 hpi, stabilizing from 24 to 48hpi. While the mutant viruses L43A, L43P and W16A showed a sloping increment similar to the one of the wt, the viruses L15A, R19A and R35A showed a slower increment in the replication (smaller slope).

Nevertheless, the PFU titers show that all of the studied viruses bearing mutations at the highlighted positions exhibit a negative impact in viral replication (*p* < 0.05) from T18 hpi onwards, with the exception of L43P virus at T24. Although the substitution of leucine (L) by alanine (A) at position 43 did not affect viral replication, replacing leucine with proline (P) at the same site resulted in a marked decrease in viral replication, similar to the effects observed in the other mutants. This difference likely reflects the distinct physicochemical properties of the substituted residues: alanine is a small, non-polar aa causing minimal structural disruption, whereas proline imposes conformational constraints due to its rigid cyclic structure, often inducing kinks in protein backbones and thereby significantly altering local protein folding and function. From the tested positions, the R35A was the mutated virus with a decreased replication profile compared to wt and the other mutated viruses (*p* < 0.05 at T18, T36 and T48), showing lower PFU titers with a slower increment.

### 3.4. Did the Combinations of Mutations Have a Cumulative Effect on Replication?

**Figure 4:**
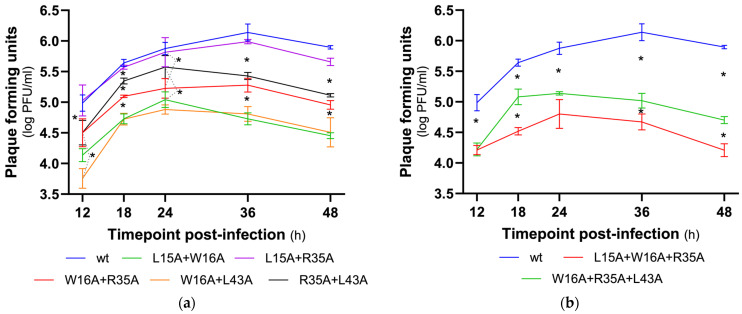
Kinetic replication curves of wt, double- mutated (**a**) and triple- mutated (**b**) viruses. The infectious viral titer (PFU) was determined by avicel plaque assay at each time point post-infection (from T12 to T48) in four independent assays (*n* = 4). All values are expressed in log PFU/mL. Asterisks indicate statistically significant differences (*p* < 0.05 Tukey).

We also analyzed the effect of double and triple mutations on viral replication kinetics (Figure 4 and Supplemental Data Table S2). The results show that, except for the double mutant L15A + R35A, all double and triple mutants had a lower replication curve than the wt at all time points (*p* < 0.05, except R35A + L43A at T24).

From the double mutants, we highlight the L15A + W16A and W16A + L43A that showed the lower replication curves, with statistical significance at T18, T36 and T48 (and at T12 for the W16A + L43A). From the triple mutants, the mutant virus L15A + W16A + R35A presented the lower kinetic curve (*p* < 0.05 for T18, T36 and T48).

Comparing the effect of the single to double mutation on the replication kinetics (Table 1), we observed contrasting results. On the one hand, we observed that the conjugation of the mutations L15A with W16A and W16A with L43A had a synergistic effect (the double mutation showed a more defective replication than the single mutation). On the other hand, conjugation of the L15A and R35A mutations had the opposite effect in the double mutant, with increased replication observed in the double mutant compared to the single mutants carrying these mutations.

The contrasting effects observed upon combining mutations at positions L15 and R35 suggest a complex interplay between these residues in modulating viral fitness. While individual mutations at these sites impair replication, their simultaneous alteration may induce compensatory structural or functional changes that partially restore or even enhance viral fitness. It is plausible that the double mutation L15A + R35A alters the NS1-RBD conformation or its interaction network in a manner that alleviates the detrimental effects caused by each single mutation alone. Such epistatic interactions are well-documented in viral proteins, where certain combinations of mutations can confer improved stability or functional adaptability, ultimately contributing to better replication capacity despite individual deleterious mutations.

Regarding the replication of the triple mutants, L15A + W16A + R35A and W16A + R35A + L43A, we observed that the former showed a reduced replication compared to the double mutants carrying the same mutations (Table 2). Indeed, the L15A + W16A + R35A mutant showed a defective replication compared to the corresponding double mutants at T18 (*p* < 0.05). This was also observed for the later time points, but without statistical significance for the L15A + W16A mutant.

With a different tendency, the triple mutant W16A + R35A + L43A shows replicative values similar to the double mutant W16A + R35A, higher than the double mutant W16A + L43A at T12 and T18 (*p* < 0.05) and lower than the double mutant R35A + L43A (*p* < 0.05 for T18–T48).

We can infer that the L15A and W16A mutations has the most significant impact on reducing viral replication. In contrast, the L43A mutation, combined with these mutations, does not decrease viral replication; rather, by introducing greater flexibility into the local protein structure, this alteration may even enhance viral fitness.

To summarize, the mutants with the greatest negative effect on viral replication were the double mutants L15A + W16A, W16A + L43A and the triple mutant L15A + W16A + R35A.

### 3.5. NA and HA Activity Profiles

**Figure 5:**
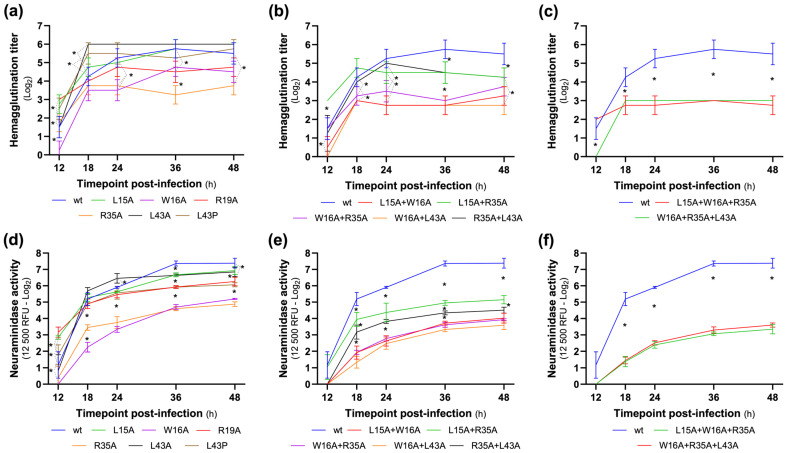
Hemagglutination titer (**a**–**c**) and Neuraminidase activity (**d**–**f**) of the wt, single (**a**,**d**), double (**b**,**e**) and triple (**c**,**f**) mutants at each time point post-infection (T12 to T48), in four independent assays (*n* = 4). The Hemagglutination titer was determined with guinea pig red blood cells and it is expressed in log_2_. The neuraminidase activity was determined by a fluorescence MUNANA-based assay, a 12,500 rfu cutoff value was used as a reference and the dilution at which the recombinant viruses achieve this neuraminidase activity was then determined. Asterisks indicate statistically significant differences (*p* < 0.05 Tukey).

In the same supernatants, we also assessed whether the mutations in the studied aa influenced HA titers and NA activity using these measurements as complementary, indirect readouts of viral replication and particle production in order to corroborate PFU-based measurements of viral fitness across NS1-RBD mutants (Figure 5 and Supplemental Data Tables S2–S6). In regard to HA titer, we observed that, among the single mutants, only the R35A mutant had a consistent defective profile compared to the wt (*p* < 0.05) from 24 hpi onwards. Two other mutants show a defective value for HA titer, but only at a certain time point, the W16A mutant at 24 hpi and the R19A mutant at 36 hpi. Unexpectedly, the L43A mutant showed a higher HA titer than the wt at 18 hpi (*p* < 0.05).

When analyzing the profiles of the double mutants, the double mutant W16A + L43A was the only one that showed a defective HA titer profile for all time points compared to the wt. The double mutants L15A + W16A and W16A + R35A showed a defective HA titer profile from 18 hpi. In addition, when compared to the respective single mutant’s HA profile, we observed that the combination of the L15A/W16A and W16A/L43A mutations showed a synergistic effect at a later stage of infection (36 and 48 hpi), since these two double mutants showed a lower HA titer than the corresponding single mutants at these time points (*p* < 0.05). On the other hand, the double mutants L15A + R35A and R35A + L43A produced higher HA titers than the R35A single mutant.

The two triple mutants also showed a defective HA titer, but without a synergistic effect, since the values obtained were similar to those obtained for the most defective double mutants.

The neuraminidase activity results showed a more defined distribution of mutants. With statistically significant (*p* < 0.05) values lower than the wt, the single mutants with the most defective NA activity were the W16A and R35A mutants, followed by R19A and L43P at 36 and 48 hpi and by L15A and L43A at 36 hpi. Regarding the double mutants, all presented a defective NA activity profile when compared with the wt (T18–T48; *p* < 0.05). Similar to the HA titer, the double mutants with the most defective NA activity were the L15A + W16A, W16A + R35A and W16A + L43A double mutants, followed by R35A + L43A and by L15A + R35A. For the W16A + L43A double mutant, a defective synergistic effect on NA activity was observed from 18 hpi onwards, with this mutant showing a statistically significant lower NA activity than the corresponding single mutants (annex). This effect was also observed for the double mutants L15A + W16A (T24–T48) and W16A + R35A (T36–T48). Both triple mutants studied showed similar defective NA activity profiles (compared to wt, *p* < 0.05); however, only the L15A + W16A + R35A showed a synergistic effect compared to the respective double mutants (and, consequently, to the respective single mutants).

Furthermore, substitutions R35A and W16A exerted the most pronounced negative effect on the production of HA and NA, given by the HA titer and NA activity; while the alteration of aa 35 affects the CDP1, the alteration of the aa 16 affects the 3 CDPs. The double mutants carrying the mutation at position 35 did not show a significant defective profile; in contrast, the double mutants carrying the mutation at position 16 presented a more defective profile than that observed for the single mutants.

## 4. Discussion

The NS1 protein of the influenza A virus is a promising antiviral target, given its multifunctionality, its conservation between subtypes, and its central role in viral replication and pathogenesis and in antagonizing the host’s innate immune responses. Previous studies, including the ones from our group, have focused predominantly on the NS1-ED (known by its role in evading host innate immunity), identifying CDPs and T-RHS, and validating their importance in viral replication through targeted mutagenesis of key residues [15,16]. Building upon this rationale, the present study shifts attention to the NS1-RBD, considering its conservation and role in viral fitness, aiming to assess whether similarly conserved and druggable sites in this domain could likewise represent vulnerable points for antiviral intervention. Although all mutations in this study were introduced into the NS1-RBD of full-length protein, potential inter-domain interaction effects can still be captured in the phenotypic analyses.

Initially, our in silico conservation and druggability integrated study identified T-RHS residues in the NS1-RBD. Single, double, and triple alanine substitutions at 5 T-RHS—L15, W16, R19, R35, and L43—were subsequently introduced into recombinant A(H1N1)pdm09 viruses, and the resulting mutants were analyzed for replication kinetics, HA capacity, and NA activity.

To our knowledge, no published study to date has specifically addressed the conservation and druggability of the NS1 protein across such a wide range of full-length NS1 sequences and crystallographic structures from human-infecting influenza subtypes, coupled with the experimental validation of NS1-RBD loss-of-function studies. This comprehensive approach, integrating extensive in silico analysis with robust experimental validation, represents a significant advance in identifying and characterizing novel antiviral targets within the NS1-RBD.

### 4.1. Single Mutations and Structural Insights

We demonstrated that specific mutations within NS1-RBD T-RHS negatively impacted viral replication to varying extents, with R35A and W16A producing the most pronounced impairment in PFU across time points. Notably, W16A localizes within a region overlapping all three described RBD-CDPs from the in silico studies, suggesting a broader structural and functional relevance of this residue. Conversely, while the mutation of L43 to alanine did not significantly affect replication, its substitution by proline (L43P) markedly impaired viral growth, consistent with the disruptive structural impact of proline on α-helical motifs. The assessment of HA titers and NA activity further supported these findings. Residues W16 and R35 consistently emerged as critical for efficient glycoprotein function, with their mutation leading to significant reduction in HA and NA activity. These phenotypic alterations may reflect upstream effects on viral replication, or, alternatively, impaired viral assembly, release, or glycoprotein incorporation.

At structural and functional levels, the residues examined here cluster within the structured six-helical bundle of the RBD dimer, which mediates high-affinity RNA binding (important for viral replication) and is also essential for innate immune evasion [42,43]. Specifically, W16 lies within helix α-1 and α-1’ and is deeply buried in the hydrophobic core of the RBD, making its side chain critical for maintaining the structural integrity of the monomer, including during the conformational transitions that occur upon dsRNA binding [44]. Consequently, the replacement of this bulky hydrophobic residue with alanine likely perturbs RBD folding, which may indirectly disrupt dimer stability or the allosteric interactions necessary for efficient RNA recognition. Similarly, R35 is surface-exposed, but highly conserved and situated adjacent to the RNA-interacting surface [13,22,45]. It likely stabilizes the electrostatic interactions with RNAs. Mutation to alanine would therefore abrogate these contacts, leading to impaired RNA sequestration and consequent loss of NS1 functionality. Among the identified interface residues, R19 plays a dual role in supporting NS1-RBD function. It facilitates the formation of the helix–helix dimer through hydrogen bonding with D39 and provides indirect structural support to the RNA-binding residue R35 [46,47]. In contrast, residues such as L15 appear to contribute more broadly to the hydrophobic integrity of the dimer interface; however, it is worth noting that direct experimental evidence for the specific contributions of some residues remains limited. The deleterious effects observed for L15A and R19A mutants reinforce their putative role in stabilizing local helicity and/or mediating indirect allosteric regulation of RNA binding. Interestingly, L43 mutation had minimal effect when replaced by alanine, but was detrimental when substituted by proline. This observation highlights the structural sensitivity of the RBD helical topology, as proline is known to induce kinks in α-helices and disrupt hydrogen bonding networks, and is consistent with previous work showing that a nearby proline substitution at position 42 (S42P) in NS1 similarly attenuates viral replication and virulence [48,49]. These results collectively validate the importance of precise side chain chemistry in maintaining RBD structural integrity and function.

### 4.2. Multiple Mutations and Structural Insights

Beyond single mutations, combinations of mutations revealed evidence of both synergistic and compensatory epistatic interactions. The double mutants L15A + W16A and W16A + L43A, as well as the triple mutant L15A + W16A + R35A, displayed significantly impaired replication compared to their corresponding single mutants (along with a reduction in HA titer and NA activity), suggesting that the cumulative disruption of structurally proximal or functionally interdependent residues amplifies the deleterious phenotype and compromises overall protein functionality, possibly by altering folding kinetics or impairing cooperative interactions between NS1 and its ligands [50]. It is still worth emphasizing that the double and triple mutants involving W16A exhibited a more severe impairment in HA/NA function than any single mutant, corroborating a model in which W16 plays a central structural role in maintaining RBD integrity and, by extension, overall viral fitness.

Conversely, the L15A + R35A double mutant paradoxically exhibited a partial rescue of replication fitness, despite each single mutation being individually deleterious. This unexpected result likely reflects a compensatory conformational effect (a phenomenon well documented in viral proteins, where epistatic interactions can buffer individual mutational costs) that stabilizes an alternative functional configuration [51,52,53]. For instance, similar epistatic buffering phenomena have been reported in other viral systems, including HIV-1 and SARS-CoV-2, and warrant further structural modelling or biophysical validation in NS1 [51,52,53].

### 4.3. NS1-RBD as a Key Antiviral Target and Future Directions

This study advances our group’s established structure-guided antiviral strategy, which leverages comprehensive sequence analysis and structural characterization to predict highly conserved druggable regions within viral proteins [15,16,54,55]. Structure-guided approaches significantly enhance research output and reduce research and development costs by promoting robust target identification, thus preventing resources from being allocated to undruggable or unreliable targets [55,56,57]. While previous work focused on the NS1-ED [15,16], this study specifically addresses the NS1-RBD of influenza A viruses, a domain whose structure-to-function relationship and antiviral potential have been underexplored.

The integration of conservation, druggability, and functional mutagenesis provides a robust framework for antiviral target prioritization. It is worth recognizing that influenza virus is able to cross the species barrier and infect humans. In this way, a NS1 protein with distinct genetic features may emerge and render a variable region within the NS1 protein that unexpectedly differs among the current IAV strains circulating in the human population. Notwithstanding, considering (i) the inherent conservation of the protein, (ii) the fact that NS1 is not under significant selective pressure, and that (iii) the predicted target regions were recognized to be highly conserved across the human-infecting IAV subtypes, during the evolution of the virus in the human host, we anticipate the virus’ difficulty in generating new NS1 mutants regarding these highly conserved sites and in developing resistance to future design-compounds targeting these regions. Our findings provide the proof-of-concept that structure-based computational predictions are invaluable for pinpointing antiviral targets and streamlining experimental efforts toward the most promising sites. The demonstration that mutations in conserved and druggable NS1-RBD residues significantly impair the A(H1N1)pdm09 virus’ replication in vitro underscores the clinical applicability of targeting the IAV NS1-RBD. In fact, our data validate the NS1-RBD as a critical functional domain harbouring conserved and druggable sites whose disruption significantly compromises viral replication. Notably, W16 and R35 map to regions of the NS1-RBD under low selective pressure, exhibiting high conservation across decades of circulating strains [16]. Targeting such evolutionarily constrained sites offers greater resilience against antiviral resistance, a critical consideration given influenza’s high mutation rate and rapid antigenic evolution. The NS1 protein’s absence from the virion, high expression during infection, and central role in antagonizing host immune responses [58], coupled with our current findings, reinforce its viability as an antiviral target. Unlike the traditional focus on NA or the viral polymerase complex, the high conservation and structural druggability of NS1, particularly in the RBD, make it an attractive candidate for small-molecule or peptide-based inhibitors. NS1’s pleiotropic roles—from inhibiting host mRNA processing [59] to modulating apoptosis and host immune responses [60]—reinforce that RBD-directed antivirals could broadly suppress viral fitness, independent of other therapeutic classes. Deciphering these molecular pathways underpinning viral replication attenuation in the NS1-RBD through altered residues is crucial. Overall, we anticipate that identifying NS1 inhibitors targeting these prioritized hot spots will yield significant clinical and pharmacological relevance. Hence, these promising NS1-RBD hot spots should be promptly explored for drug design of new ligands or drug discovery using virtual screening, an efficient method for guiding the search for potent molecules. Such molecules, potentially offering higher resilience to viral resistance, could represent a new class of broad-spectrum antiviral drugs, mitigating seasonal influenza epidemics and future pandemics.

Within this NS1-centred framework, the present work provides functional validation of in silico-prioritized NS1-RBD hot spots using a robust in vitro influenza A virus replication system. While this approach is appropriate to sensitively detect mutation-specific effects on viral replication and fitness, it does not recapitulate the full complexity of in vivo infection or the contribution of innate immune responses and other host factors, and the results should therefore be interpreted as an initial step towards more comprehensive translational evaluation. In this regard, these findings represent a critical first step that warrants further validation in more complex systems, such as air–liquid interface models or in vivo studies.

Future studies should also aim to resolve high-resolution structures of the RBD in complex with host or RNA ligands to inform the rational design of inhibitory molecules targeting the validated hot spots identified herein. This work also sets the stage for future mechanistic studies to resolve the structural impact of these mutations via crystallography or cryo-electron microscopy. In vivo validation and resistance profiling will be essential to establish the therapeutic relevance of these targets and their potential robustness against viral escape.

Ultimately, in line with the WHO Global Influenza Strategy 2019–2030 [4], which calls for innovative antiviral strategies beyond current NA inhibitors, our results reaffirm the NS1 protein as a viable and underexplored therapeutic target and bridge the gap between basic and translational research in anti-NS1 influenza strategies. As emphasized, these findings open new avenues for NS1-targeted antiviral discovery and development, potentially enabling broader-spectrum and resistance-resilient influenza therapeutics.

## Figures and Tables

**Table 1:** Comparison of replication between the single and the double mutants.

Timepoint	T12			T18			T24			T36			T48		
Mutants	single^(a)^	double^(a)^	Δ (*p*)^(b)^	single^(a)^	double^(a)^	Δ (*p*)^(b)^	single^(a)^	double^(a)^	Δ (*p*)^(b)^	single^(a)^	double^(a)^	Δ (*p*)^(b)^	single^(a)^	double^(a)^	Δ (*p*)^(b)^
L15A	5.02 ± 0.11	4.14 ± 0.10	↓(<0.01)	5.38 ± 0.10	4.73 ± 0.08	↓(<0.01)	5.51 ± 0.10	5.04 ± 0.13	↓(<0.01)	5.60 ± 0.10	4.73 ± 0.10	↓(<0.01)	5.47 ± 0.15	4.45 ± 0.05	↓(<0.01)
W16A	4.39 ± 0.28		↓(0.61)	5.10 ± 0.02		↓(<0.01)	5.22 ± 0.10		↓(0.91)	5.37 ± 0.15		↓(<0.01)	5.25 ± 0.17		↓(<0.01)
L15A	5.02 ± 0.11	5.03 ± 0.25	↑(1)	5.38 ± 0.10	5.57 ± 0.03	↑(0.049)	5.51 ± 0.10	5.82 ± 0.24	↑(0.18)	5.60 ± 0.10	5.99 ± 0.04	↑(<0.01)	5.47 ± 0.15	5.66 ± 0.06	↑(0.59)
R35A	4.58 ± 0.04		↑(0.02)	4.83 ± 0.11		↑(<0.01)	5.01 ± 0.08		↑(<0.01)	4.99 ± 0.09		↑(<0.01)	4.76 ± 0.14		↑(<0.01)
W16A	4.39 ± 0.28	4.50 ± 0.23	↑(1)	5.10 ± 0.02	5.10 ± 0.02	↓(1)	5.22 ± 0.10	5.23 ± 0.16	↑(1)	5.37 ± 0.15	5.28 ± 0.11	↓(1)	5.25 ± 0.17	4.96 ± 0.07	↓(0.05)
R35A	4.58 ± 0.04		↓(1)	4.83 ± 0.11		↑(<0.01)	5.01 ± 0.08		↑(0.67)	4.99 ± 0.09		↑(0.047)	4.76 ± 0.14		↑(0.52)
W16A	4.39 ± 0.28	3.76 ± 0.16	↓(<0.01)	5.10 ± 0.02	4.72 ± 0.10	↓(<0.01)	5.22 ± 0.10	4.88 ± 0.07	↓(0.10)	5.37 ± 0.15	4.81 ± 0.12	↓(<0.01)	5.25 ± 0.17	4.51 ± 0.24	↓(<0.01)
L43A	4.98 ± 0.08		↓(<0.01)	5.61 ± 0.08		↓(<0.01)	5.95 ± 0.06		↓(<0.01)	5.94 ± 0.11		↓(<0.01)	5.87 ± 0.17		↓(<0.01)
R35A	4.58 ± 0.04	4.50 ± 0.20	↓(1)	4.83 ± 0.11	5.35 ± 0.05	↑(<0.01)	5.01 ± 0.08	5.57 ± 0.19	↑(<0.01)	4.99 ± 0.09	5.43 ± 0.06	↑(<0.01)	4.76 ± 0.14	5.11 ± 0.03	↑(0.01)
L43A	4.98 ± 0.08		↓(0.01)	5.61 ± 0.08		↓(<0.01)	5.95 ± 0.06		↓(0.03)	5.94 ± 0.11		↓(<0.01)	5.87 ± 0.17		↓(<0.01)

^(a)^ Mean of log PFU/mL ± standard deviation for single and double mutants. ^(b)^ Difference between single and double mutants with *p* in brackets. The increase and decrease in replication from the single to the double mutant are indicated by an up (↑) and down (↓) arrow, respectively. Increases and decreases that are statistically significant (*p* < 0.05) are highlighted in red and green, respectively.

**Table 2:** Comparison of replication between the double and the triple mutants.

Timepoint	T12			T18			T24			T36			T48		
**Mutants**	**double^(a)^**	**triple^(a)^**	**Δ (*p*)^(b)^**	**double^(a)^**	**triple^(a)^**	**Δ (*p*)^(b)^**	**double^(a)^**	**triple^(a)^**	**Δ (*p*)^(b)^**	**double^(a)^**	**triple^(a)^**	**Δ (*p*)^(b)^**	**double^(a)^**	**triple^(a)^**	**Δ (*p*)^(b)^**
L15A+	4.14 ± 0.10	4.21 ± 0.08	↑(1)	4.73 ± 0.08	4.52 ± 0.06	**↓(0.02)**	5.04 ± 0.13	4.80 ± 0.24	↓(0.53)	4.73 ± 0.10	4.67 ± 0.13	↓(1)	4.45 ± 0.05	4.21 ± 0.10	↓(0.20)
W16A															
L15A+	5.03 ± 0.25		**↓(<0.01)**	5.57 ± 0.03		**↓(<0.01)**	5.82 ± 0.24		**↓(<0.01)**	5.99 ± 0.04		**↓(<0.01)**	5.66 ± 0.06		**↓(<0.01)**
R35A															
W16A+	4.50 ± 0.23		↓(0.42)	5.10 ± 0.02		**↓(<0.01)**	5.23 ± 0.16		**↓(0.01)**	5.28 ± 0.11		**↓(<0.01)**	4.96 ± 0.07		**↓(<0.01)**
R35A															
W16A+	4.50 ± 0.23	4.22 ± 0.11	↓(0.47)	5.10 ± 0.02	5.08 ± 0.13	↓(1)	5.23 ± 0.16	5.14 ± 0.03	↓(1)	5.28 ± 0.11	5.02 ± 0.12	↓(0.11)	4.96 ± 0.07	4.7 ± 0.06	↓(0.16)
R35A															
W16A+	3.76 ± 0.16		**↑(0.01)**	4.72 ± 0.10		**↑(<0.01)**	4.88 ± 0.07		↑(0.44)	4.81 ± 0.12		↑(0.35)	4.51 ± 0.24		↑(0.55)
L43A															
R35A+	4.50 ± 0.20		↓(0.45)	5.35 ± 0.05		**↓(<0.01)**	5.57 ± 0.19		**↓(<0.01)**	5.43 ± 0.06		**↓(<0.01)**	5.11 ± 0.03		**↓(<0.01)**
L43A															

^(a)^ Mean of log PFU/mL ± standard deviation for double and the triple mutants. ^(b)^ Difference between double mutants and the corresponding triple mutant (L15A + W16A + R35A—top row and W16A + R35A + L43A—bottom row) with *p* in parenthesis. The increase and decrease in replication from the double to the respective triple mutant are indicated by an up (↑) and down (↓) arrow, respectively. Increases and decreases that are statistically significant (*p* < 0.05) are highlighted in red and green, respectively.

## Data Availability

The original contributions presented in this study are included in the article/Supplementary Material. Further inquiries can be directed to the corresponding author.

## Supplementary Materials

The following supporting information can be downloaded at https://www.mdpi.com/article/10.3390/v18030279/s1. Table S1. Overall alignment of NS1-RBD druggability prediction, Table S2. Comparison of PFU titration, Hemagglutination titer and Neuraminidase activity between the wild-type and the mutants, Table S3. Comparison of Hemagglutination titer between the single and the double mutants, Table S4. Comparison of Hemagglutination titer between the double and the corresponding triple mutants, Table S5. Comparison of Neuraminidase activity between the single and the double mutants, Table S6. Comparison of Neuraminidase activity between the double and the corresponding triple mutants.

## Abbreviations

The following abbreviations are used in this manuscript:

aa	amino acid
CDP	Conserved Druggable Pocket
CDP1	Consensus Druggable Pocket 1
CDP2	Consensus Druggable Pocket 2
CDP3	Consensus Druggable Pocket 3
DGSS	DoGSiteScorer
ED	Effector Domain
HA	Hemagglutination
hpi	hours post-infection
MDCK-SIAT1	Madin Darby Canine Kidney cells with overexpression of the α-2,6-linked sialic acid receptor
MOE	Molecular Operating Environment
MOI	Multiplicity Of Infection
MUNANA	2′-(4-Methylumbelliferyl)-α-D-N-acetylneuraminic acid sodium salt hydrate
NA	Neuraminidase
NMR	Nuclear Magnetic Resonance
NS1	Non-Structural protein 1
NS1-ED	NS1 Effector Domain
NS1-RBD	NS1 RNA-Binding Domain
PDS	PockDrug-Server
PFU	Plaque-Forming Units
RBD	RNA-Binding Domain
RFU	Relative Fluorescence Units
SVM	Support Vector Machine
T-RHS	Top-Ranked Hot Spot (T-RHS)
wt	Wild-type
