# Supplementary material for: The RNA-Binding Domain NS1 of Influenza as an Antiviral Target: From Evolutionary Conservation Mapping to Experimental Validation
Source: Viruses. 2026 Feb 25;18(3):279. doi: 10.3390/v18030279 (PMC13029851; doi:10.3390/v18030279)
Supplement: Supplementary file 1 [file viruses-18-00279-s001.zip › viruses-4129004-supplementary.pdf]

Supplementary Data

Table S1 – Overall alignment of NS1-RBD druggability prediction. Druggability prediction based on estimation by the pocket bioinformatic tools – PockDrug-Server (PDS) and DoGSiteScorer (DGSS) using the NS1-RBD dimeric

			1	2	3	4	5	6	7	8	9	10	11
1NS1	DIMER	POCKDRUG	−	−	−	−	−	−	+	+	+	+	+
		DOGSITESCORER	−	−	−	−	+	+	+	+	+	+	+
2N74	DIMER	POCKDRUG	−	−	−	−	+	−	−	+	+	+	+
		DOGSITESCORER	−	−	−	+	+	−	+	+	+	+	+
2ZKO	DIMER	POCKDRUG	−	−	−	−	−	−	−	−	−	−	−
		DOGSITESCORER	−	−	−	−	+	−	−	+	+	−	+
3M8A	DIMER	POCKDRUG	−	−	−	−	+	−	+	+	+	−	−
		DOGSITESCORER	−	−	−	+	+	−	−	+	+	−	+
		Druggability score				0-2	5-6	0-2	3-4	7-8	7-8	3-4	5-6

			12	13	14	15	16	17	18	19	20	21	22
1NS1	DIMER	POCKDRUG	+	+	+	+	+	+	+	+	+	+	+
		DOGSITESCORER	+	+	+	+	+	+	+	+	−	+	+
2N74	DIMER	POCKDRUG	−	−	+	+	+	+	+	+	+	+	+
		DOGSITESCORER	+	+	+	+	+	+	+	+	+	+	+
2ZKO	DIMER	POCKDRUG	+	+	+	+	+	+	+	+	+	+	−
		DOGSITESCORER	+	+	+	+	+	+	+	+	+	+	−
3M8A	DIMER	POCKDRUG	+	+	+	+	+	+	+	+	−	+	+
		DOGSITESCORER	+	+	+	+	+	+	+	+	+	+	+
		Druggability score	7-8	7-8	7-8	7-8	7-8	7-8	7-8	7-8	5-6	7-8	5-6

			23	24	25	26	27	28	29	30	31	32	33
1NS1	DIMER	POCKDRUG	+	+	+	−	+	−	+	−	+	+	−
		DOGSITESCORER	−	−	+	−	+	−	+	+	+	+	−
2N74	DIMER	POCKDRUG	+	+	+	+	+	+	+	+	+	+	+
		DOGSITESCORER	+	−	+	+	+	+	+	+	+	+	+
2ZKO	DIMER	POCKDRUG	−	+	−	−	−	−	−	−	−	+	−
		DOGSITESCORER	−	−	−	−	−	+	+	+	+	+	−
3M8A	DIMER	POCKDRUG	−	−	+	+	+	−	+	−	+	+	−
		DOGSITESCORER	−	−	+	−	+	−	+	−	+	+	+
		Druggability score	3-4	3-4	5-6	3-4	5-6	3-4	7-8	3-4	7-8	7-8	3-4

			34	35	36	37	38	39	40	41	42	43	44
1NS1	DIMER	POCKDRUG	−	+	+	−	−	+	−	−	+	+	−
		DOGSITESCORER	−	+	+	−	+	+	+	+	+	+	+
2N74	DIMER	POCKDRUG	−	+	+	−	−	+	+	−	−	+	+
		DOGSITESCORER	−	+	+	−	−	+	+	−	−	+	+
2ZKO	DIMER	POCKDRUG	−	+	+	−	−	+	+	−	+	+	+
		DOGSITESCORER	−	+	+	−	+	+	+	+	+	+	+
3M8A	DIMER	POCKDRUG	−	+	+	−	+	+	+	+	+	+	+
		DOGSITESCORER	+	+	−	−	−	+	+	−	+	+	+
		Druggability score	0-2	7-8	7-8	0-2	3-4	7-8	7-8	3-4	5-6	7-8	7-8

			45	46	47	48	49	50	51	52	53	54	55
1NS1	DIMER	POCKDRUG	−	+	−	−	−	−	−	−	−	+	+
		DOGSITESCORER	−	+	+	−	−	+	+	+	+	+	+
2N74	DIMER	POCKDRUG	−	+	+	+	−	+	+	+	+	+	+
		DOGSITESCORER	−	+	+	−	−	+	−	−	−	+	+
2ZKO	DIMER	POCKDRUG	−	+	+	+	−	−	+	+	+	+	+
		DOGSITESCORER	+	+	−	−	+	+	−	−	−	+	+
3M8A	DIMER	POCKDRUG	+	+	+	+	+	+	+	+	+	+	+
		DOGSITESCORER	−	+	−	−	+	+	−	−	−	+	+
		Druggability score	0-2	7-8	5-6	3-4	3-4	5-6	3-4	3-4	3-4	7-8	7-8

			56	57	58	59	60	61	62	63	64	65	66
1NS1	DIMER	POCKDRUG	−	−	+	+	−	+	+	+	+	+	+
		DOGSITESCORER	+	+	+	+	+	+	+	+	+	+	+
2N74	DIMER	POCKDRUG	−	−	+	+	−	−	+	−	−	+	+
		DOGSITESCORER	+	−	+	+	+	−	+	−	+	+	+
2ZKO	DIMER	POCKDRUG	+	−	+	+	−	−	+	+	−	+	+
		DOGSITESCORER	−	−	+	−	−	−	+	−	−	+	+
3M8A	DIMER	POCKDRUG	+	−	+	−	−	−	+	+	+	−	−
		DOGSITESCORER	−	−	+	+	−	−	+	+	+	−	−
		Druggability score	3-4	0-2	7-8	5-6	0-2	0-2	7-8	5-6	5-6	5-6	5-6

			67	68	69	70	71	72	73
1NS1	DIMER	POCKDRUG	+	+	+	−	+	+	+
		DOGSITESCORER	−	+	+	−	−	+	+
2N74	DIMER	POCKDRUG	−	−	+	+	−	−	−
		DOGSITESCORER	−	+	+	+	−	−	−
2ZKO	DIMER	POCKDRUG	−	−	+	+	−	−	−
		DOGSITESCORER	+	−	+	−	−	−	−
3M8A	DIMER	POCKDRUG	+	+	+	+	+	−	−
		DOGSITESCORER	+	+	+	+	+	−	−
		Druggability score	3-4	5-6	7-8	5-6	3-4	0-2	0-2

Druggability score scale 0-2 | 3-4 | 5-6 | 7-8

Table S2 - Comparison of PFU titration, Hemagglutination titer and Neuraminidase activity between the wild-type and the mutants

	Timepoint	T12			T18			T24			T36			T48		
	Mutants	*wt*	[a]	Δ (*p*)[b]	*wt*	[a]	Δ (*p*)[b]	*wt*	[a]	Δ (*p*)[b]	*wt*	[a]	Δ (*p*)[b]	*wt*	[a]	Δ (*p*)[b]
PFU Titration	L15A		5.02±0.11	↑ (1)		5.38±0.1	↓ (<0.01)		5.51±0.1	↓ (0.047)		5.6±0.1	↓ (<0.01)		5.47±0.15	↓ (<0.01)
	W16A		4.39±0.28	↓ (<0.01)		5.1±0.02	↓ (<0.01)		5.22±0.1	↓ (<0.01)		5.37±0.15	↓ (<0.01)		5.25±0.17	↓ (<0.01)
	R19A		5.05±0.11	↑ (1)		5.17±0.05	↓ (<0.01)		5.42±0.21	↓ (<0.01)		5.63±0.09	↓ (<0.01)		5.37±0.04	↓ (<0.01)
	R35A		4.58±0.04	↓ (0.046)		4.83±0.11	↓ (<0.01)		5.01±0.08	↓ (<0.01)		4.99±0.09	↓ (<0.01)		4.76±0.14	↓ (<0.01)
	L43A		4.98±0.08	↓ (1)		5.61±0.08	↓ (1)		5.95±0.06	↑ (1)		5.94±0.11	↓ (0.42)		5.87±0.17	↓ (1)
	L43P		4.65±0.17	↓ (0.22)		5.27±0.09	↓ (<0.01)		5.54±0.11	↓ (0.09)		5.54±0.17	↓ (<0.01)		5.11±0.09	↓ (<0.01)
	L15A+W16A	4.99±0.13	4.14±0.1	↓ (<0.01)	5.64±0.06	4.73±0.08	↓ (<0.01)	5.88±0.1	5.04±0.13	↓ (<0.01)	6.14±0.14	4.73±0.1	↓ (<0.01)	5.9±0.03	4.45±0.05	↓ (<0.01)
	L15A+R35A		5.03±0.25	↑ (1)		5.57±0.03	↓ (0.99)		5.82±0.24	↓ (1)		5.99±0.04	↓ (0.83)		5.66±0.06	↓ (0.25)
	W16A+R35A		4.5±0.23	↓ (<0.01)		5.1±0.02	↓ (<0.01)		5.23±0.16	↓ (<0.01)		5.28±0.11	↓ (<0.01)		4.96±0.07	↓ (<0.01)
	W16A+L43A		3.76±0.16	↓ (<0.01)		4.72±0.1	↓ (<0.01)		4.88±0.07	↓ (<0.01)		4.81±0.12	↓ (<0.01)		4.51±0.24	↓ (<0.01)
	R35A+L43A		4.5±0.2	↓ (<0.01)		5.35±0.05	↓ (<0.01)		5.57±0.19	↓ (0.2)		5.43±0.06	↓ (<0.01)		5.11±0.03	↓ (<0.01)
	L15A+W16A+R35A		4.21±0.08	↓ (<0.01)		4.52±0.06	↓ (<0.01)		4.8±0.24	↓ (<0.01)		4.67±0.13	↓ (<0.01)		4.21±0.1	↓ (<0.01)
	W16A+R35A+L43A		4.22±0.11	↓ (<0.01)		5.08±0.13	↓ (<0.01)		5.14±0.03	↓ (<0.01)		5.02±0.12	↓ (<0.01)		4.7±0.06	↓ (<0.01)
Hemagglutination Titer	L15A		2.75±0.5	↑ (0.04)		4.75±0.5	↑ (0.86)		5±0	↑ (1)		5.75±0.5	= (1)		5.5±0.58	= (1)
	W16A		0.25±0.5	↓ (0.04)		3.5±0.58	↓ (0.32)		3.5±0.58	↓ (<0.01)		4.75±0.5	↓ (0.12)		4.5±0.58	↓ (0.2)
	R19A		3±0	↑ (<0.01)		4±0	↑ (1)		4.75±0.5	↓ (0.94)		4.5±0.58	↓ (0.02)		4.75±0.5	↓ (0.63)
	R35A		1.75±0.5	↑ (1)		3.75±0.5	↓ (0.865)		3.75±0.5	↓ (<0.01)		3.25±0.5	↓ (<0.01)		3.75±0.5	↓ (<0.01)
	L43A		1.5±0.58	↓ (1)		6±0	↑ (<0.01)		6±0	↑ (0.53)		6±0	↑ (1)		6±0	↑ (0.97)
	L43P		2.5±0.58	↑ (0.22)		5.5±0.58	↑ (<0.01)		5.5±0.58	↑ (1)		5.25±0.5	↓ (0.94)		5.75±0.5	↑ (1)
	L15A+W16A	1.5±0.58	0.5±0.58	↓ (0.22)	4.25±0.5	3±0	↓ (<0.01)	5.25±0.5	2.75±0.5	↓ (<0.01)	5.75±0.5	2.75±0.5	↓ (<0.01)	5.5±0.58	3.25±0.5	↓ (<0.01)
	L15A+R35A		3±0	↑ (<0.01)		4.75±0.5	↑ (0.86)		4.5±0.58	↓ (0.53)		4.5±0.58	↓ (0.02)		4.25±0.5	↓ (0.04)
	W16A+R35A		1.5±0.58	↓ (1)		3.25±0.5	↓ (0.045)		3.5±0.58	↓ (<0.01)		3±0	↓ (<0.01)		3.75±0.5	↓ (<0.01)
	W16A+L43A		0±0	↓ (<0.01)		3±0	↓ (<0.01)		2.75±0.5	↓ (<0.01)		2.75±0.5	↓ (<0.01)		2.75±0.5	↓ (<0.01)
	R35A+L43A		1.25±0.96	↓ (1)		4±0	↑ (1)		5±0	↑ (1)		4.5±0.58	↓ (0.02)		4.25±0.5	↓ (0.04)
	L15A+W16A+R35A		2±0	↑ (0.97)		2.75±0.5	↓ (<0.01)		2.75±0.5	↓ (<0.01)		3±0	↓ (<0.01)		2.75±0.5	↓ (<0.01)
	W16A+R35A+L43A		0±0	↓ (<0.01)		3±0	↓ (<0.01)		3±0	↓ (<0.01)		3±0	↓ (<0.01)		3±0	↓ (<0.01)
Neuraminidase Activity	L15A		2.83±0.11	↑ (<0.01)		5.28±0.25	↑ (1)		5.63±0.06	↓ (0.978)		6.67±0.12	↓ (<0.01)		6.93±0.24	↓ (0.242)
	W16A		0±0	↓ (0.06)		2.25±0.29	↓ (<0.01)		3.33±0.18	↓ (<0.01)		4.7±0.15	↓ (<0.01)		5.2±0.03	↓ (<0.01)
	R19A		3.15±0.33	↑ (<0.01)		4.9±0.3	↓ (0.98)		5.46±0.2	↓ (0.59)		5.92±0.08	↓ (<0.01)		6.26±0.26	↓ (<0.01)
	R35A		0.38±0.7	↓ (0.525)		3.45±0.17	↓ (<0.01)		3.75±0.36	↓ (<0.01)		4.61±0.13	↓ (<0.01)		4.88±0.14	↓ (<0.01)
	L43A		0.82±0.98	↓ (1)		5.69±0.2	↑ (0.53)		6.45±0.29	↑ (0.25)		6.63±0.08	↓ (<0.01)		6.85±0.29	↓ (0.08)
	L43P		1.79±0.6	↑ (0.83)		4.88±0.08	↓ (0.97)		5.57±0.39	↓ (0.9)		5.91±0.07	↓ (<0.01)		6.06±0.12	↓ (<0.01)
	L15A+W16A	1.17±0.8	0±0	↓ (0.06)	5.19±0.4	1.93±0.4	↓ (<0.01)	5.9±0.06	2.63±0.32	↓ (<0.01)	7.36±0.16	3.71±0.12	↓ (<0.01)	7.38±0.3	4.02±0.3	↓ (<0.01)
	L15A+R35A		1.04±0.75	↓ (1)		3.94±0.43	↓ (<0.01)		4.37±0.57	↓ (<0.01)		4.95±0.15	↓ (<0.01)		5.15±0.26	↓ (<0.01)
	W16A+R35A		0±0	↓ (0.06)		1.93±0.13	↓ (<0.01)		2.77±0.15	↓ (<0.01)		3.62±0.14	↓ (<0.01)		3.93±0.05	↓ (<0.01)
	W16A+L43A		0±0	↓ (0.06)		1.34±0.36	↓ (<0.01)		2.47±0.35	↓ (<0.01)		3.33±0.13	↓ (<0.01)		3.59±0.26	↓ (<0.01)
	R35A+L43A		0±0	↓ (0.06)		3.17±0.43	↓ (<0.01)		3.82±0.12	↓ (<0.01)		4.35±0.13	↓ (<0.01)		4.51±0.18	↓ (<0.01)
	L15A+W16A+R35A		0±0	↓ (0.06)		1.38±0.31	↓ (<0.01)		2.39±0.2	↓ (<0.01)		3.07±0.12	↓ (<0.01)		3.35±0.29	↓ (<0.01)
	W16A+R35A+L43A		0±0	↓ (0.06)		1.45±0.2	↓ (<0.01)		2.52±0.14	↓ (<0.01)		3.3±0.19	↓ (<0.01)		3.6±0.13	↓ (<0.01)

[a] Mean of log PFU/ml ± standard deviation (PFU titration); Mean of log_2 HA ± standard deviation (Hemagglutination titer); Mean of log_2 NA ± standard deviation (Neuraminidase activity)

[b] Difference between mutants and the *wild-type* with *p* in brackets. The increase and decrease in replication from the *wild-type* to the mutant are indicated by an up (↑) and down (↓) arrow, respectively. Increases and decreases that are statistically significant (p<0.05) are highlighted in **red** and **green**, respectively.

Table S3 - Comparison of Hemagglutination titer between the single and the double mutants

Timepoint	T12			T18			T24			T36			T48		
Mutants	single[a]	double[a]	Δ (*p*)[b]	single[a]	double[a]	Δ (*p*)[b]	single[a]	double[a]	Δ (*p*)[b]	single[a]	double[a]	Δ (*p*)[b]	single[a]	double[a]	Δ (*p*)[b]
L15A	2.75±0.50	0.50±0.58	↓ (<0.01)	4.75±0.50	3.00±0.00	↓ (<0.01)	5.00±0.00	2.75±0.50	↓ (<0.01)	5.75±0.50	2.75±0.50	↓ (<0.01)	5.50±0.58	3.25±0.50	↓ (<0.01)
W16A	0.25±0.50		↑ (1)	3.50±0.58		↑ (0.87)	3.50±0.58		↓ (0.53)	4.75±0.50		↓ (<0.01)	4.50±0.58		↓ (0.04)
L15A	2.75±0.50	3.00±0.00	↑ (1)	4.75±0.50	4.75±0.50	- (1)	5.00±0.00	4.50±0.58	↓ (0.94)	5.75±0.50	4.5±0.58	↓ (0.02)	5.50±0.58	4.25±0.50	↓ (0.04)
R35A	1.75±0.50		↑ (0.04)	3.75±0.50		↑ (0.045)	3.75±0.50		↑ (0.53)	3.25±0.50		↑ (0.02)	3.75±0.50		↑ (0.97)
W16A	0.25±0.50	1.50±0.58	↑ (0.04)	3.50±0.58	3.25±0.50	↓ (1)	3.50±0.58	3.50±0.58	- (1)	4.75±0.50	3.00±0.00	↓ (<0.01)	4.50±0.58	3.75±0.50	↓ (0.63)
R35A	1.75±0.50		↓ (1)	3.75±0.50		↓ (0.87)	3.75±0.50		↓ (1)	3.25±0.50		↑ (1)	3.75±0.50		- (1)
W16A	0.25±0.50	0.00±0.00	↑ (1)	3.50±0.58	3.00±0.00	↑ (0.87)	3.50±0.58	2.75±0.50	↓ (0.53)	4.75±0.50	2.75±0.50	↓ (<0.01)	4.50±0.58	2.75±0.50	↓ (<0.01)
L43A	1.50±0.58		↓ (<0.01)	6.00±0.00		↓ (<0.01)	6.00±0.00		↓ (<0.01)	6.00±0.00		↓ (<0.01)	6.00±0.00		↓ (<0.01)
R35A	1.75±0.50	1.25±0.96	↓ (0.97)	3.75±0.50	4.00±0.00	↑ (1)	3.75±0.50	5.00±0.00	↑ (0.02)	3.25±0.50	4.50±0.58	↑ (0.02)	3.75±0.50	4.25±0.50	↑ (0.97)
L43A	1.50±0.58		↓ (1)	6.00±0.00		↓ (<0.01)	6.00±0.00		↓ (0.13)	6.00±0.00		↓ (<0.01)	6.00±0.00		↓ (<0.01)

[a] Mean of log_2 HA ± standard deviation for single and double mutants

[b] Difference between single and double mutants with *p* in brackets. The increase and decrease in replication from the single to the double mutant are indicated by an up (↑) and down (↓) arrow, respectively. Increases and decreases that are statistically significant (*p*<0.05) are highlighted in **red** and **green**, respectively.

Table S4 - Comparison of Hemagglutination titer between the double and the corresponding triple mutants

Timepoint	T12			T18			T24			T36			T48		
Mutants	double[a]	triple[a]	Δ (*p*)[b]	double[a]	triple[a]	Δ (*p*)[b]	double[a]	triple[a]	Δ (*p*)[b]	double[a]	triple[a]	Δ (*p*)[b]	double[a]	triple[a]	Δ (*p*)[b]
**L15A+ W16A**	0.50±0.58		**↑ (<0.01)**	3.00±0.00		↓ (1)	2.75±0.50		↓ (1)	2.75±0.50		↑ (1)	3.25±0.50		↓ (0.97)
**L15A+ R35A**	3.00±0.00	2.00±0.00	↓ (0.22)	4.75±0.50	2.75±0.50	**↓ (<0.01)**	4.50±0.58	2.75±0.50	**↓ (<0.01)**	4.50±0.58	3.00±0.00	**↓ (<0.01)**	4.25±0.50	2.75±0.50	**↓ (<0.01)**
**W16A+ R35A**	1.50±0.58		↑ (0.97)	3.25±0.50		↓ (0.87)	3.50±0.58		↓ (0.53)	3.00±0.00		↓ (1)	3.75±0.50		↓ (0.20)
**W16A+ R35A**	1.50±0.58		**↓ (<0.01)**	3.25±0.50		↑ (1)	3.50±0.58		↑ (0.94)	3.00±0.00		↓ (1)	3.75±0.50		↓ (0.63)
**W16A+ L43A**	0.00±0.00	0.00±0.00	- (1)	3.00±0.00	3.00±0.00	- (1)	2.75±0.50	3.00±0.00	↑ (1)	2.75±0.50	3.00±0.00	↑ (1)	2.75±0.50	3.00±0.00	↑ (1)
**R35A+ L43A**	1.25±0.96		**↓ (0.04)**	4.00±0.00		**↓ (0.05)**	5.00±0.00		**↓ (<0.01)**	4.50±0.58		**↓ (<0,02)**	4.25±0.50		**↓ (0.04)**

[a] Mean of log_2 HA ± standard deviation for double and triple mutants

[b] Difference between double and triple mutants with *p* in brackets. The increase and decrease in replication from the double to the triple mutant are indicated by an up (↑) and down (↓) arrow, respectively. Increases and decreases that are statistically significant (p<0.05) are highlighted in **red** and **green**, respectively.

Table S5 - Comparison of Neuraminidase activity between the single and the double mutants

Timepoint	T12			T18			T24			T36			T48		
Mutants	single[a]	double[a]	Δ (*p*)[b]	single[a]	double[a]	Δ (*p*)[b]	single[a]	double[a]	Δ (*p*)[b]	single[a]	double[a]	Δ (*p*)[b]	single[a]	double[a]	Δ (*p*)[b]
**L15A**	2.83±0.11	0.00±0.00	**↓ (<0.01)**	5.28±0.25	1.93±0.40	**↓ (<0.01)**	5.63±0.06	2.63±0.32	**↓ (<0.01)**	6.67±0.12	3.71±0.12	**↓ (<0.01)**	6.93±0.24	4.02±0.30	**↓ (<0.01)**
**W16A**	0.00±0.00		- (1)	2.25±0.29		↓ (0.96)	3.33±0.18		**↓ (0.049)**	4.70±0.15		**↓ (<0.01)**	5.20±0.03		**↓ (<0.01)**
**L15A**	2.83±0.11	1.04±0.75	**↓ (<0.01)**	5.28±0.25	3.94±0.43	**↓ (<0.01)**	5.63±0.06	4.37±0.57	**↓ (<0.01)**	6.67±0.12	4.95±0.15	**↓ (<0.01)**	6.93±0.24	5.15±0.26	**↓ (<0.01)**
**R35A**	0.38±0.70		↑ (0.77)	3.45±0.17		↑ (0.56)	3.75±0.36		↑ (0.13)	4.61±0.13		**↑ (0.03)** (red)	4.88±0.14		↑ (0.89)
**W16A**	0.00±0.00	0.00±0.00	- (1)	2.25±0.29	1.93±0.13	↓ (0.96)	3.33±0.18	2.77±0.15	↓ (0.24)	4.70±0.15	3.62±0.14	**↓ (<0.01)**	5.20±0.03	3.93±0.05	**↓ (<0.01)**
**R35A**	0.38±0.70		↓ (1)	3.45±0.17		**↓ (<0.01)**	3.75±0.36		**↓ (<0.01)**	4.61±0.13		**↓ (<0.01)**	4.88±0.14		**↓ (<0.01)**
**W16A**	0.00±0.00	0.00±0.00	- (1)	2.25±0.29	1.34±0.36	**↓ (<0.01)**	3.33±0.18	2.47±0.35	**↓ (<0.01)**	4.70±0.15	3.33±0.13	**↓ (<0.01)**	5.20±0.03	3.59±0.26	**↓ (<0.01)**
**L43A**	0.82±0.98		↓ (0.47)	5.69±0.20		**↓ (<0.01)**	6.45±0.29		**↓ (<0.01)**	6.63±0.08		**↓ (<0.01)**	6.85±0.29		**↓ (<0.01)**
**R35A**	0.38±0.70	0.00±0.00	↓ (1)	3.45±0.17	3.17±0.43	↓ (0.99)	3.75±0.36	3.82±0.12	↑ (1)	4.61±0.13	4.35±0.13	↓ (0.26)	4.88±0.14	4.51±0.18	↓ (0.56)
**L43A**	0.82±0.98		↓ (0.47)	5.69±0.20		**↓ (<0.01)**	6.45±0.29		**↓ (<0.01)**	6.63±0.08		**↓ (<0.01)**	6.85±0.29		**↓ (<0.01)**

[a] Mean of log_2 NA ± standard deviation for single and double mutants

[b] Difference between single and double mutants with *p* in brackets. The increase and decrease in replication from the single to the double mutant are indicated by an up (↑) and down (↓) arrow, respectively. Increases and decreases that are statistically significant (p<0.05) are highlighted in **red** and **green**, respectively.

Table S6 - Comparison of Neuraminidase activity between the double and the corresponding triple mutants

Timepoint	T12			T18			T24			T36			T48		
Mutants	double[a]	triple[a]	Δ (*p*)[b]	double[a]	triple[a]	Δ (*p*)[b]	double[a]	triple[a]	Δ (*p*)[b]	double[a]	triple[a]	Δ (*p*)[b]	double[a]	triple[a]	Δ (*p*)[b]
**L15A+ W16A**	0.00±0.00		- (1)	1.93±0.40		↓ (0.40)	2.63±0.32		↓ (0.99)	3.71±0.12		**↓ (<0.01)**	4.02±0.30		**↓ (0.01)**
**L15A+ R35A**	1.04±0.75	0.00±0.00	↓ (0.14)	3.94±0.43	1.38±0.31	**↓ (<0.01)**	4.37±0.57	2.39±0.20	**↓ (<0.01)**	4.95±0.15	3.07±0.12	**↓ (<0.01)**	5.15±0.26	3.35±0.29	**↓ (<0.01)**
**W16A+ R35A**	0.00±0.00		- (1)	1.93±0.13		↓ (0.39)	2.77±0.15		↓ (0.78)	3.62±0.14		**↓ (<0.01)**	3.93±0.05		**↓ (0.04)**
**W16A+ R35A**	0.00±0.00		- (1)	1.93±0.13		↓ (0.60)	2.77±0.15		↓ (0.99)	3.62±0.14		↓ (0.07)	3.93±0.05		↓ (0.73)
**W16A+ L43A**	0.00±0.00	0.00±0.00	- (1)	1.34±0.36	1.45±0.20	↑ (1)	2.47±0.35	2.52±0.14	↑ (1)	3.33±0.13	3.30±0.19	↓ (1)	3.59±0.26	3.60±0.13	↑ (1)
**R35A+ L43A**	0.00±0.00		- (1)	3.17±0.43		**↓ (<0.01)**	3.82±0.12		**↓ (<0.01)**	4.35±0.13		**↓ (<0.01)**	4.51±0.18		**↓ (<0.01)**

[a] Mean of log_2 NA ± standard deviation for double and triple mutants

[b] Difference between double and triple mutants with *p* in brackets. The increase and decrease in replication from the double to the triple mutant are indicated by an up (↑) and down (↓) arrow, respectively. Increases and decreases that are statistically significant (p<0.05) are highlighted in **red** and **green**, respectively.
